# Development and implementation of a pharmacy PrEP awareness-raising and referral pathway: acceptability and feasibility of a UK pilot

**DOI:** 10.1136/sextrans-2025-056711

**Published:** 2026-02-11

**Authors:** China Harrison, Lindsey Harryman, Sarah Stockwell, Hannah Family, Joanna May Kesten, Sarah Denford, Jennifer Scott, Caroline Sabin, Joanna Copping, John Saunders, Ross Hamilton-Shaw, Natalie Symonds, Osarenoma B Dick, Emma Tarmey, Jeremy Horwood

**Affiliations:** 1 National Institute for Health and Care Research Applied Research Collaboration West (NIHR ARC West), Bristol, UK; 2 National Institute for Health and Care Research, Health Protection Research Unit (HPRU) in Evaluation and Behavioural Science (EBS), Bristol, UK; 3 Population Health Sciences, University of Bristol, Bristol, UK; 4 University Hospitals Bristol and Weston (UHBW), NHS Foundation Trust, Bristol, UK; 5 Department of Sexual Health, Cardiff Royal Infirmary, Cardiff, UK; 6 Institute for Global Health, UCL, London, UK; 7 National Institute for Health and Care Research (NIHR) Health Protection Research Unit (HPRU) in Blood-Borne and Sexually Transmitted Infections, University College London, London, UK; 8 Communities and Public Health, Bristol City Council, Bristol, UK; 9 Blood Safety, Hepatitis, STI & HIV Division, UK Health Security Agency, London, UK; 10 Gilead Sciences Ltd, London, UK; 11 Public Health and Wellbeing, South Gloucestershire Council, South Gloucestershire, UK; 12 Patient and Public Involvement Collaborator, Bristol, UK; 13 Patient and Public Involvement Contributor, Bristol, UK

**Keywords:** PrEP, HIV, sexual health

## Abstract

**Abstract:**

**Objectives:**

To develop and pilot a community pharmacy pre-exposure prophylaxis (PrEP) awareness-raising and referral pathway, informed by a literature review and interviews with pharmacists and community members.

**Methods:**

Using the person-based approach and the Capability, Opportunity, Motivation-Behaviour change (COM-B) model, the pilot aimed to improve HIV/PrEP knowledge/capability and motivation through pharmacist and community awareness-raising. Opportunity was addressed by integrating PrEP consultations into pharmacy services and offering sexually transmitted infection (STI), HIV and kidney function home self-sampling kits. Financial incentives aimed at motivating pharmacists. Pharmacists initiated PrEP discussions and provided self-sampling kits. Individuals interested in starting PrEP were referred to the sexual health clinic, completed the tests and posted them to the clinic for processing. Clinic staff then confirmed PrEP eligibility and prescribed PrEP remotely. National Health Service (NHS) policy restrictions on pharmacies stocking NHS-procured PrEP informed the delivery model. The pilot evaluation focused on staff training impact, number/type of consultations and acceptability and feasibility for staff and community members.

**Results:**

The pilot was conducted in five pharmacies across Bristol, North Somerset and South Gloucestershire from October 2024 to April 2025. A total of 55 individuals (aged 18–69 years, n=23 male, n=31 female, n=1 transgender) were made aware of PrEP and offered a referral for PrEP. Visit reasons included emergency contraception (n=14), PrEP (n=14) and opioid substitution therapy (n=11). Twenty-eight community members expressed interest in PrEP; 22 accepted a self-sampling kit, nine returned it and four were prescribed PrEP. Pharmacists and community members viewed the service positively. Barriers included pharmacists’ initial lack of confidence initiating PrEP discussions and limited public awareness of PrEP availability in pharmacies.

**Conclusion:**

Community pharmacies represent a promising site for a PrEP awareness-raising and referral pathway. Successful scale-up requires pharmacist training, public health education and structural and policy changes to support accessible PrEP provision beyond sexual health clinics to reduce inequities.

**WHAT IS ALREADY KNOWN ON THIS TOPIC**

**WHAT THIS STUDY ADDS**

- This is the first UK pilot of a community pharmacy-based PrEP awareness-raising and referral pathway, targeting underserved community members.
- The approach was found to be acceptable and feasible for both pharmacists and community members, highlighting the role of pharmacies as potentially valuable settings to raise awareness of PrEP and support access beyond traditional sexual health clinics.

**HOW THIS STUDY MIGHT AFFECT RESEARCH, PRACTICE OR POLICY**

- Pharmacy-based PrEP delivery requires targeted training, supportive policy frameworks and sustainable funding to enable effective provision outside of sexual health clinics.

## Introduction

 Pre-exposure prophylaxis (PrEP) is a highly effective HIV prevention method^1^ that significantly reduces new HIV acquisitions. In the UK, PrEP is available free of charge from publicly funded sexual health services. Those initiating PrEP undergo baseline and regular follow-up screening tests for sexually transmitted infections (STIs), HIV and kidney function.^2^ Consequently, PrEP is primarily accessed via face-to-face clinic-based services.

However, reliance on clinic-based provision has raised concerns about inequitable access. Populations for whom HIV acquisition remains high but PrEP use is low (eg, communities of black African and Caribbean heritage, people who inject drugs (PWID), women and transgender people), face particular challenges, contributing to persistent health inequalities.^1 3 4^ Previous research has highlighted barriers such as limited awareness of PrEP, geographical distance from sexual health clinics and stigma, particularly within communities where sexual health discussions may be culturally taboo^5^.^6–10^

In response, the UK government, national PrEP guidelines and various research studies have proposed the expansion of PrEP provision to community pharmacies and other settings to enhance awareness, accessibility and uptake.^11–14^ Pharmacies are widely available in urban and rural areas, have extended working hours and may feel less stigmatising than sexual health clinics.^7 10^ They are well placed to deliver consultations and support PrEP initiation because they are experienced in providing diverse health information and services (eg, Pharmacy First) including a range of sexual health services. However, despite their favourable positioning, pharmacies face structural and capacity-related constraints, including staff workload, staff shortages and limited consultation space, which could affect their ability to take on additional PrEP responsibilities.^7 10^

Numerous international studies have demonstrated the effectiveness of pharmacy-based PrEP awareness-raising and initiation, particularly for underserved populations,^11 15^ and our previous research has highlighted the acceptability of hypothetical pharmacy-based PrEP awareness-raising and initiation in the UK.^7 10^ This study aimed to develop, implement and pilot a UK pharmacy-based PrEP awareness-raising and referral pathway for PrEP initiation at a sexual health clinic (hereafter intervention) and examine its acceptability and feasibility. The intervention was developed to target underserved community members not currently aware of and/or accessing PrEP.

## Methods and materials

A mixed-methods design was used to develop and evaluate the intervention.

### Pharmacy PrEP intervention development

The intervention design was guided by the person-based approach (PBA)^16^ behaviour change theory and mixed methods research. The PBA is an iterative framework for intervention development incorporating evidence, and stakeholder and patient and public involvement (PPI) input (figure 1). The first stage involved identifying the barriers and facilitators to pharmacy PrEP delivery using the Capability, Opportunity, Motivation, Behaviour (COM-B) model of behaviour change.^7 10 17^ The second stage involved coproducing the pilot design and promotional materials with community members, supported by three stakeholder workshops (pharmacists, sexual health clinicians, local authority commissioners) and two PPI meetings with four men (n=1) and women (n=3) of diverse ethnicities (black African, white British) and sexual orientations (heterosexual, bisexual).

**Figure 1:**
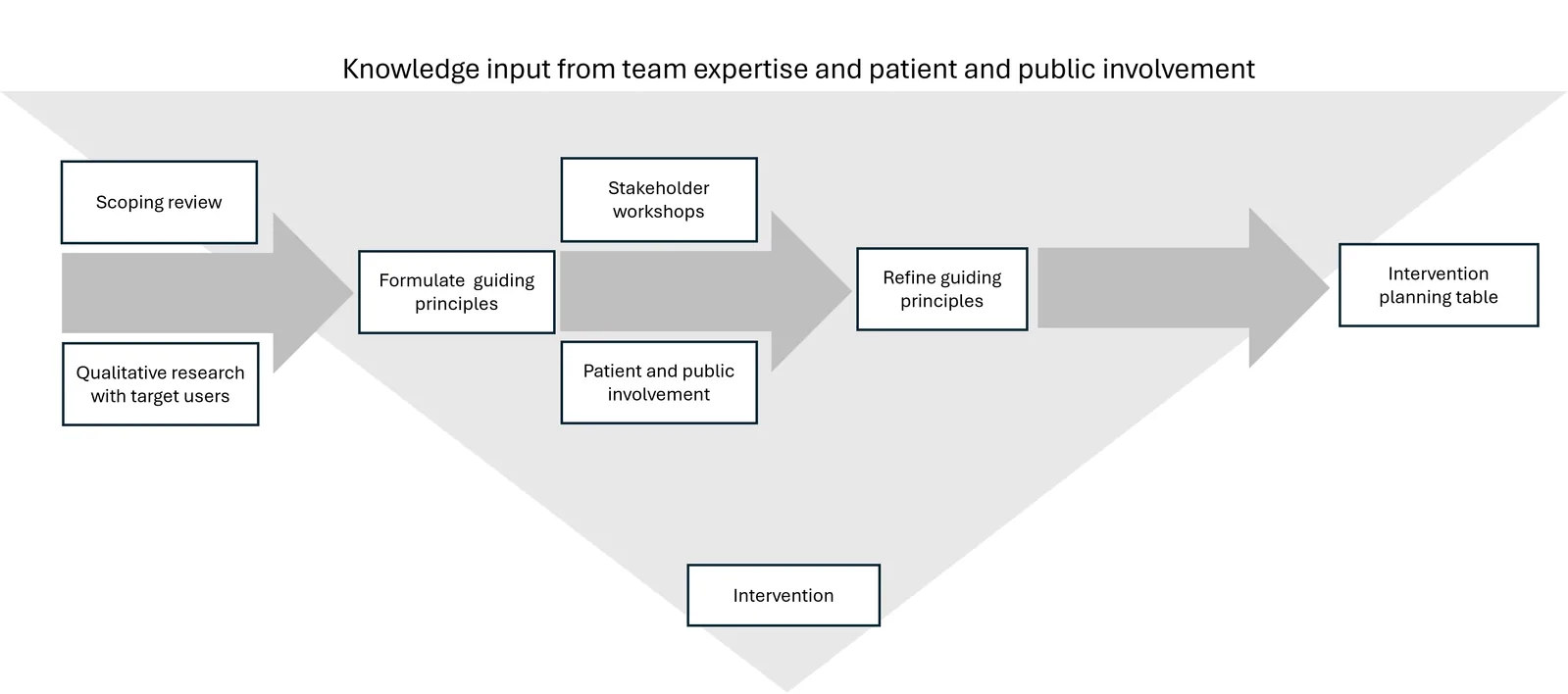
Person-based approach study design overview.

The identified barriers and facilitators^7 10^ were collated with stakeholders and PPI input (positive and negative) to create the PBA guiding principles and intervention planning table (figure 1).^16^ This iterative process pointed to the need to enhance capabilities and motivation through staff training and community awareness raising. Additional strategies included increasing access opportunities by integrating PrEP discussions into pre-existing pharmacy services and offering home STI (gonorrhoea, chlamydia, syphilis), hepatitis B and C, HIV and kidney function (estimated glomerular filtration rate) self-sampling kits. Because pharmacies were unable to procure or stock affordable PrEP via National Health Service (NHS) England medicines procurement and supply chain, the intervention was designed in collaboration with a sexual health clinic to enable pharmacists to raise awareness of PrEP and provide community members with a referral pathway to the sexual health clinic for remote PrEP initiation. To further support motivation, pharmacies received £1000 for set up and training and £30 (in line with similar services) per PrEP awareness-raising consultation (hereafter consultation) recorded in PharmOutcomes (a secure computer clinical service platform).

### Pharmacy PrEP intervention design

All pharmacy staff completed online HIV/PrEP training adapted from Terrence Higgins Trust.^18^ Pharmacists also attended two in-person training sessions delivered by the research team and sexual health clinicians. These sessions addressed questions, enhanced knowledge and supported pharmacists to initiate PrEP discussions through peer learning and practice across consultation scenarios (eg, emergency contraception, opioid substitution). The first in-person session occurred prior to the pilot’s launch, the second 3 months later. The first author (CH) regularly visited the pharmacies to support intervention delivery.

To raise community awareness, posters were displayed in the pilot pharmacies, community spaces (eg, notice boards, doctors’ surgeries) and organisations (eg, Bristol Drugs Project, Refugee Women of Bristol). Digital posters were shared online via community organisations and social media. Paid Facebook and Instagram advertisements ran from 14 to 23 February and 3 to 4 March 2025. Promotional material directed individuals to the five pharmacies and the pilot website.

The intervention consisted of seven steps (figure 2):

1. Community members either attend the pharmacy to discuss PrEP or are made aware of PrEP by the pharmacist.
2. A PrEP consultation takes place in a private consultation room with a pharmacist who gives an overview of PrEP, its effectiveness, who it is for, how to use the self-sampling kits and the next steps for accessing PrEP.
3. The community member decides whether to be referred to the sexual health clinic to initiate PrEP.
4. If the community member wishes to proceed, the pharmacist provides them with a home self-sampling kit and sends an electronic referral to the sexual health clinic.
5. The community member completes the self-sampling kit at home and sends it to the sexual health clinic for processing in the lab.
6. When results are available, the sexual health clinic prescriber phones the community member and has a remote consultation to provide test results, determine PrEP suitability, discuss safety, dosing regimen and ongoing supply from the sexual health clinic.
7. If the community member is eligible and wishes to proceed, the sexual health clinic prescribes and posts PrEP to the community member’s home. The community member then enters routine sexual health service pathways for ongoing PrEP supply.

**Figure 2:**
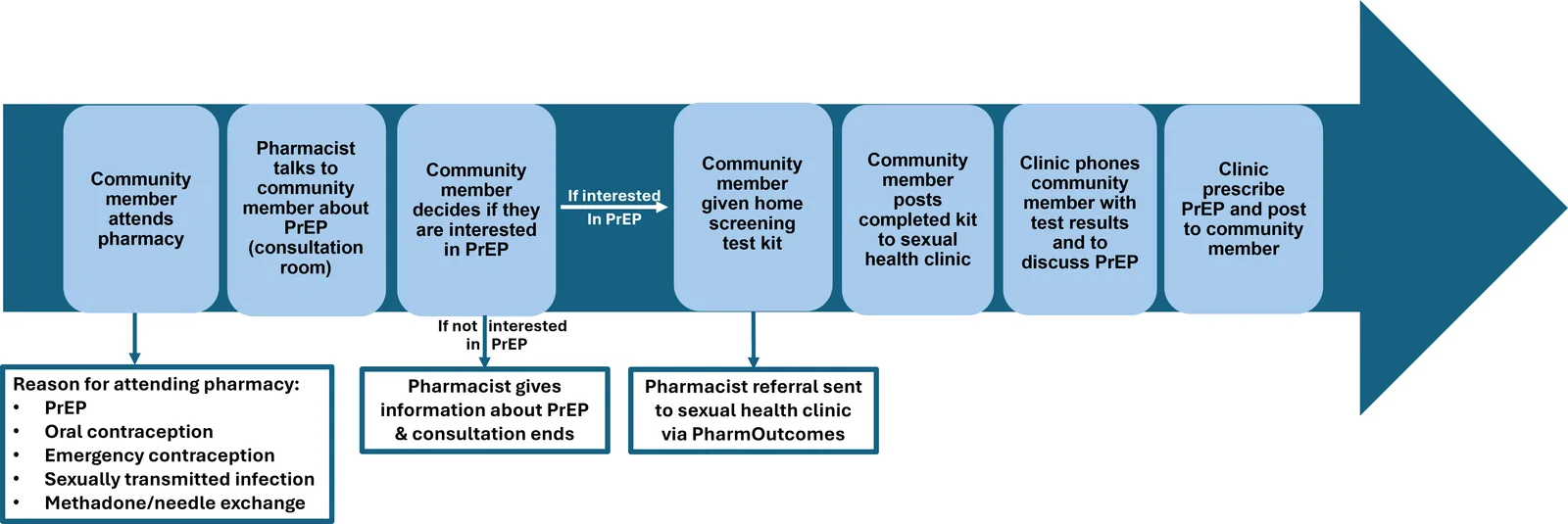
Pharmacy pre-exposure prophylaxis (PrEP) awareness-raising and referral pathway intervention flow diagram.

Pharmacists used PharmOutcomes to guide them through what to discuss, record consultations and electronically refer community members to the sexual health clinic (step 4). Referrals included the community members’ contact details (name, phone number), so the clinic was aware of who had a consultation and could contact them to discuss test results, PrEP and arrange PrEP delivery.

### Study setting

The intervention was implemented in five pharmacies across Bristol (n=3), North Somerset (n=1) and South Gloucestershire (n=1) (BNSSG) between October 2024 and April 2025. Pharmacies were invited by the Local Pharmaceutical Committee and selected to maximise variation in HIV prevalence, community ethnic diversity, rural/urban location and number of sexual health-related consultations provided.

### Study population

Community members were eligible to participate if they were ≥18 years, HIV negative, not currently using PrEP and able to consent. All pharmacy staff at the five pharmacies were eligible to complete the online training and pharmacists delivering PrEP consultations were eligible for interviews.

### Quantitative evaluation methods

#### Training evaluation

To assess training impact, all pharmacy staff completed a 10-question HIV and PrEP knowledge quiz immediately before and after completing the online training.^18^ Participants were asked to rate statements as true, false or do not know. Correct answers were scored one point, while incorrect or do not know answers scored zero points. After completion, the correct answers for any questions answered incorrectly were provided.

#### Consultations and initiations

The PrEP PharmOutcomes template was designed specifically to enable pharmacists to record participant demographics (eg, age, gender, ethnicity, phone number), reason for attending the pharmacy, interest in starting PrEP, whether a self-sampling kit was provided, if not why not and to enable an electronic referral to be sent to the sexual health clinic.

The sexual health clinic recorded the number of self-sampling kits returned and PrEP prescriptions initiated, along with participants’ age, gender and ethnicity.

#### Qualitative evaluation

Interviews were conducted with the pharmacists at months 3 and 6 to explore implementation, acceptability and feasibility. Pharmacists were invited via email by CH, provided with a participant information sheet and offered a £40 shopping voucher for participation.

After their PrEP consultation, community members were invited to complete a survey online (via QR code) or on paper about their experiences, by the pharmacists. At the end of the survey, participants could provide their email to receive information about taking part in an interview. The sexual health clinic also sent a text message invitation to those referred from pharmacies for PrEP who had not yet completed the survey. Interested participants were provided with a participant information sheet and invited to an interview. Community members received a £20 shopping voucher for participation.

### Data analysis

Quantitative data were analysed using SPSS. A Wilcoxon signed-rank test assessed difference between pre-training and post-training knowledge scores. Descriptive statistics (means (M), SD and percentage) summarised PrEP consultations and community member demographics.

Interviews lasting 30–45 min were conducted online, by telephone or in person by CH using flexible topic guides exploring views and experiences of the intervention. Interviews were audio recorded, transcribed, imported into NVivo and analysed thematically.^19^ Transcripts were read several times for familiarisation, then coded inductively and deductively line-by-line to capture participants’ views and experiences. Community members and pharmacists’ data were analysed separately and concurrently, then triangulated with quantitative data to provide a more nuanced understanding. Data were coded by CH and reviewed by HF to sense check their interpretation and provide an alternative perspective on the data. Themes were generated by reviewing and grouping connecting codes and discussed by the multidisciplinary team to ensure transparency, coherence and commitment to rigour.

## Results

### Staff training

All 31 customer-facing staff in the five pharmacies completed the online training. A Wilcoxon signed-rank test showed a significant immediate increase in knowledge scores from pre-training (M=4.77, SD=3.03) to post-training (M=9.10, SD=1.90; Z=−4.47, p<0.001). Ten pharmacists also completed the in-person training sessions.

### Pilot consultations and referrals

A total of 55 community members had a pharmacy PrEP consultation during the 6-month pilot. Most consultations were with females aged 20–29 years, and those of white British ethnicity (table 1). Most consultations were pharmacist-initiated when individuals attended the pharmacy for other services (n=25) including opioid substitution therapy (eg, methadone, n=11) or emergency contraception (n=14). A total of 14 community members actively approached the pharmacy to discuss PrEP.

**Table 1:** Demographic characteristics of community members (n=55) having a community pharmacy PrEP consultation, taking a self-sampling kit, returning a self-sampling kit and being prescribed PrEP

	Pharmacy PrEP consultation (n=55)	Self-sampling kits provided (n=22)	Self-sampling kits returned (n=9)	Prescribed PrEP (n=4)
**Characteristic**				
Gender n (%)				
Male	23 (41.82)	9 (40.91)	4 (44.44)	3 (75.00)
Female	31 (56.36)	12 (54.55)	5 (55.55)	1 (25.00)
Transgender	1 (1.82)	1 (4.55)		
Unknown				
Age (years) n (%)				
18–19	5 (9.09)	3 (13.64)	1 (11.11)	
20–29	17 (30.91)	9 (40.91)	3 (33.33)	2 (50.00)
30–39	11 (20.00)	6 (27.27)	3 (33.33)	
40–49	8 (14.55)	1 (4.55)		
50–59	12 (21.81)	3 (13.64)	2 (22.22)	2 (50.00)
60–69	2 (3.64)			
Ethnicity n (%)				
Asian or Asian British	2 (3.64)	1 (4.55)		
Mixed white and Asian	2 (3.64)			
Black or black British	2 (3.64)	1 (4.55)	2 (22.22)	1 (25.00)
Not stated/prefer not to say	19 (34.55)	10 (45.45)	1 (11.11)	
White British/white other	30 (34.55)	10 (45.45)	6 (66.67)	3 (75.00)

PrEP, pre-exposure prophylaxis.

Of the 55 individuals who had a PrEP consultation, 28 (51%) were interested in PrEP. Of these, 22 (88%) took a self-sampling kit and were referred to the sexual health clinic. Those not taking a self-sampling kit predominantly reported that they wanted more time to think about PrEP.

Of those referred to the clinic, nine kits were returned: one person was uncontactable, three declined PrEP and four were prescribed PrEP (table 1). Of those prescribed PrEP, three were known to the sexual health clinic due to having previously ordered postal self-sampling kits (n=2) or having visited the clinic (n=1).

### Qualitative evaluation

Seven community members and five pharmacists participated in interviews. Community members were women (n=4) and men (n=3) aged 24–54 years and identified as white (n=5) or black (n=2) ethnicity, and as bisexual (n=3), gay (n=1) or heterosexual (n=3). Three were prescribed PrEP and four declined a PrEP referral.

Analysis led to the development of key themes outlined below, reflecting the perspectives of pharmacists and community members. Illustrative quotations (box 1) are provided with participant identifiers: community member (CM), pharmacist (P), plus interview time point (T1/T2).

Box 1 **Illustrative quotes from the interviews for the four themes**

**Acceptability of pharmacy pre-exposure prophylaxis (PrEP) awareness-raising referral pathway**

*“Oh, I need to go here (sexual health clinic) in an emergency, whereas a pharmacy is more—I think it pushes people towards being preventative rather than reactive in terms of sexual health”. CM5*

*“But when it’s the local chemist, it’s so accessible. It’s really accessible, after work you can go and it’s no appointment needed, and you don’t have a long wait in a queue. So, it makes it easy, easy access really”. CM7*

**Experiences of PrEP consultations**

*“There would be the, um (-), you know, there would always be in the back of your mind ’oh, if somebody sees me here, they’re going to ask questions”. CM3*

*“I was quite nervous (to attend a sexual health clinic). I felt anxious and I suppose there was a level of sort of guilt”. CM5*

*“The interaction I had (with pharmacists) was very, very straightforward. It wasn’t intimidating at all. The pharmacist was very approachable, very friendly”. CM3*

*“I think education is really important, so I didn’t mind that she brought it up, I was quite fine with it”. CM1*

“*Immediately thought it (PrEP) wasn’t something I would need, but I did think it was interesting, and just something I would have never considered before […] because I’m a Christian and realistically my goal is to not have, you know, sex before marriage*”. *CM1*

“*It wasn’t a pressing concern for me, I suppose. But knowing that the option is there, um (-), was I suppose quite reassuring*”. *CM6*

**Experiences of service delivery**

*“You felt a little bit like you were pre-judging a patient on their exploits, their sexual exploits”. P1 T2*

*“The second (training) helped (ease) feeling embarrassed or worried about offending someone when offering this service”. P3 T1*

*“So it may have been a bit of a slow start to the pilot, but once we’d done a few and became a bit more familiar with what was happening, what it was all about, then it seemed to flow a little bit better”. P5 T2*

*“It’s been easier with the (customers collecting) methadone because obviously we’ve got a relationship with them, generally patients that we know”. P1 T2*

*“Probably with the emergency contraception, that was the easiest one to link the two things together”. P3 T1*

*“Up to 50’s, yeah, for me I found myself quite comfortable to approach the subject with them, but over that range I thought it wasn’t really necessary”. P2 T2*

*“I think I find it a little bit difficult just to ask people to go into the consultation room just for the purposes of PrEP unless we’ve got a genuine concern that we kind of pick up in the initial conversation”. P5 P1*

**Suggested improvements**

*“I saw the poster on the wall whilst waiting, however, I didn’t even think it would be a service for me […], perhaps I wasn’t looking at the sign correctly but I saw HIV and I thought oh right, that might be for people who suffer with it”. CM6*

*“I did find that in order for the scheme to work better there should be more advertisement”. P3 T2*

*“The only problem sometimes is that I find it takes about two or three fingers [(laughs]) to get the right amount of blood in the vial and I end up coming out like a pin cushion”. CM3*

*“I think it’s a bit long that time (to wait). I think they should reduce it”. CM2*

*“We could supply the medicines and all that kind of thing then it would probably give us a lot more […] that would probably give us a bit more sort of regular contact with these people, so if we can see that they keep coming back”. P5 T2*

#### Acceptability of a pharmacy PrEP awareness-raising and referral pathway

Pharmacists and community members viewed a pharmacy PrEP awareness-raising and referral pathway positively, reporting pharmacies to be an important, beneficial option to support PrEP initiation outside of sexual health clinics. They highlighted pharmacies to be useful points of contact for preventative rather than reactive service provision. Pharmacies were also viewed positively for their accessibility, anonymity and potential for raising awareness of PrEP.

#### Experiences of PrEP consultations

Community members reported positive experiences of PrEP consultations and felt comfortable discussing PrEP with the pharmacist. Individuals who were made aware of PrEP while visiting the pharmacy for other services appreciated being informed, especially as they reported having never heard of PrEP before. Those who actively sought PrEP from the pharmacy described the process as accessible and relatively straightforward. Those who declined PrEP cited personal irrelevance due to faith, abstinence from sex, stable or monogamous relationships or preferences for behaviour change over medication.

#### Experiences of service delivery

Pharmacists delivering the intervention reported experiencing some initial challenges. These primarily related to feelings of embarrassment and unease or concern about how best to bring up the conversation of PrEP with their customers. This was exacerbated by only having a few community members attending the pharmacies specifically for PrEP and was particularly pertinent at the beginning of the pilot due to concerns about causing offence or appearing judgemental. Confidence increased after the second in-person training session and with greater experience of conducting consultations as the pilot progressed.

The pharmacist-initiated PrEP consultations were more commonly undertaken with their regular customers with whom they had pre-existing relationships, particularly those collecting methadone prescriptions. Pharmacists also found it easier to raise awareness of PrEP when providing sexual health services, for example, emergency contraception and during quieter periods, when workload pressures were lower. Turning an over-the-counter inquiry into a PrEP consultation was also reported to be challenging, as it required tactfully moving the customer into a private consultation room.

In other cases, pharmacists made quick assessments based on timing, context and customer socio-demographic characteristics to determine whether a PrEP consultation would be appropriate. They tended to avoid raising awareness of PrEP with older individuals or those in long-term relationships who were seen as less likely to benefit from being made aware of PrEP.

#### Suggested improvements

Community members and pharmacists highlighted several key areas for improvement to ensure service feasibility and effectiveness. Pharmacists highlighted the low number of customers actively attending the pharmacy for PrEP. In contrast, community members highlighted their lack of awareness of PrEP and its availability via pharmacies. Most community members reported not seeing the PrEP awareness-raising material distributed during the pilot. Those who did often overlooked them, perceiving them as not personally relevant. To address this gap, community members and pharmacists suggested targeted and widespread campaigns, via social media, television and posters. These efforts also seem to help reduce HIV and sexual health-related stigma.

Concerns about the complexity of the self-sampling kits were also raised, including lack of translated instructions, collecting the blood and delays between sampling, obtaining results and receiving PrEP. Enabling pharmacists to stock and dispense PrEP directly to community members was suggested to help streamline initiation and support ongoing relationships with customers.

## Discussion

This is the first UK pilot to explore the acceptability and feasibility of pharmacies raising awareness of PrEP and providing a referral pathway to a sexual health clinic for oral PrEP initiation, with a particular focus on underserved community members. This novel pilot suggests that pharmacies could be an acceptable and feasible route for PrEP awareness-raising and to sexual health referral. Notably, this pilot reached a high percentage of women and PWID, groups less likely to be aware of and engage with traditional PrEP services. However, it is important to acknowledge that three of the four individuals who went on to initiate PrEP were known to sexual health services, indicating that opportunities to raise awareness and promote PrEP uptake also exist within existing services, including online postal self-sampling (OPSS) pathways. This highlights the value of strengthening both pharmacy-based and clinic-based awareness-raising strategies to maximise reach. Findings also highlight the need to build pharmacist confidence, raise community awareness and reduce structural and logistical barriers to allow direct pharmacy PrEP initiation and provision to maximise reach and reduce burden on sexual health clinics.

UK community pharmacists already provide sexual and reproductive health services such as emergency contraception, chlamydia screening, condom distribution and oral contraception. The successful expansion of these protocol-driven services demonstrates pharmacists’ ability to acquire the specialist training and competencies required to deliver sexual healthcare, supporting their potential role in expanding PrEP access beyond traditional sexual health clinics.^20^ While the pilot suggests pharmacy PrEP awareness-raising and referral was acceptable, pharmacists did express initial concerns about initiating conversations about PrEP with their customers. This challenge reflects early stage difficulties in embedding new practices in routine care.^13 20^ However, as with other clinical services, pharmacists’ confidence and perceived capability improved with training and experience. Consistent with previous research, these findings underscore the importance of accessible, high-quality training and ongoing support to enable pharmacy staff to effectively raise awareness of PrEP.^7 10 21^

Limited PrEP awareness remains a key barrier to initiation.^7^ This pilot used community pharmacies’ accessibility and provision of existing sexual health and clinical services to reach groups who may otherwise be unaware of PrEP. Results suggest that expanding opportunities to engage individuals in discussions about PrEP, pharmacies could strategically improve awareness among underserved groups, particularly women and/or PWID attending for emergency contraception or opioid substitution therapy. Embedding PrEP consultations into existing services could help to routinise conversations, improve awareness and normalise PrEP use beyond gay, bisexual and other men who have sex with men communities. This could additionally help support broader system-wide approaches for tackling HIV stigma and reduce unconscious bias or selective offering of PrEP that reinforces inequalities. The finding that community members had either not seen PrEP awareness-raising materials or had not considered them personally relevant highlights the need for these efforts to be supported by local and national campaigns to raise awareness of PrEP among key groups.^22^

While community pharmacies could play a significant role in raising PrEP awareness and initiation, their inability to procure or stock affordable NHS PrEP necessitated delivery in collaboration with a sexual health clinic. In England, PrEP is funded by NHS England and commissioned through local authorities via the Public Health Grant. For this pilot, a collaborative practice agreement between five pharmacies and the local sexual health clinic was established to overcome these barriers and enable pharmacies to raise awareness of PrEP and refer interested individuals for PrEP initiation. The complexity and delays within the pathway may have caused some individuals to reconsider starting PrEP, become lost in the referral process or fail to complete self-sampling kits and initiate PrEP. For example, only 32% of the self-sampling kits provided were returned, compared with 51% for home STI self-sampling kits from vending machines and 65% for OPSS in BNSSG.^23^ Additionally, three individuals who returned their self-sampling kit subsequently declined PrEP, and one was uncontactable.

The lower return rate of self-sampling kits may reflect difficulties collecting blood samples at home required for PrEP.^7^ Future research should explore strategies such as in-person support, translated materials and private pharmacy spaces to facilitate kit completion, particularly for individuals with limited English literacy, PWID, individuals with no fixed abode or those who struggle to take their own blood. It is also possible that some individuals took a self-sampling kit but did not return it before the pilot concluded, contributing to a lower return rate. Developing pathways that enable pharmacists to raise awareness, supply and manage PrEP prescriptions autonomously could also help address these barriers, as well as prevent individuals from being lost in the complex referral pathway.

While independent prescribing pharmacists could reduce reliance on manual referrals and help streamline access, direct pharmacy PrEP provision also requires pharmacies to be able to stock NHS PrEP. Without access to NHS PrEP, costs would be prohibitively expensive for local authorities, pharmacies and customers.^7 10 21^ Providing an autonomous, free comprehensive PrEP service may become increasingly important with the introduction of long-acting injectable PrEP (ie, cabotegravir and lenacapavir), as pharmacies could support initiation and continuation of care, easing pressure on sexual health services. With cabotegravir administered every 8 weeks and lenacapavir every 6 months, community pharmacies could serve as additional points of contact. Future research should examine the acceptability and feasibility of delivering long-acting injectable PrEP through pharmacies.

### Strengths and limitations

The pilot was developed using robust PBA methods, combining literature, qualitative interviews, PPI and behavioural theory to ensure accessibility and relevance for underserved communities and interviews offered insights into the experiences and suggestions for optimisation. However, findings are from a single area and should be interpreted accordingly. While the pathway was co-designed with sexual health clinicians, staff involved in PrEP initiation were not interviewed. Future research should investigate larger, long-term pharmacy PrEP pilots, including perspectives from customers, sexual health staff and wider stakeholders, and assess delivery across pharmacies, community settings and primary care.^13^ It should also consider impacts on clinic workloads, including managing inadequate samples and the need for repeat testing.

## Conclusion

The pilot suggests community pharmacies are potentially acceptable, feasible and transformative settings for PrEP awareness-raising and referral. With appropriate training, support, policy alignment and commissioning, pharmacies could also potentially stock, supply and manage PrEP prescriptions autonomously, playing a critical role in reducing HIV transmission and sexual health inequalities.

## Data Availability

Data are available on reasonable request.
